# Insight into the mechanism and outcoupling enhancement of excimer-associated white light generation†
†Electronic supplementary information (ESI) available. CCDC 1008747. For ESI and crystallographic data in CIF or other electronic format see DOI: 10.1039/c5sc04902d


**DOI:** 10.1039/c5sc04902d

**Published:** 2016-02-12

**Authors:** Ying-Hsiao Chen, Kuo-Chun Tang, Yi-Ting Chen, Jiun-Yi Shen, Yu-Sin Wu, Shih-Hung Liu, Chun-Shu Lee, Chang-Hsuan Chen, Tzu-Yu Lai, Shih-Huang Tung, Ru-Jong Jeng, Wen-Yi Hung, Min Jiao, Chung-Chih Wu, Pi-Tai Chou

**Affiliations:** a Department of Chemistry , National Taiwan University , Taipei , 10617 Taiwan , Republic of China . Email: chop@ntu.edu.tw; b Institute of Optoelectronic Sciences , National Taiwan Ocean University , Keelung 20224 , Taiwan , Republic of China . Email: wenhung@mail.ntou.edu.tw; c Institute of Polymer Science and Engineering , National Taiwan University , Taipei , 10617 Taiwan , Republic of China . Email: rujong@ntu.edu.tw; d Department of Electrical Engineering , Graduate Institute of Electronics Engineering, and Graduate Institute of Photonics and Optoelectronics , National Taiwan University , Taipei 10617 , Taiwan , Republic of China

## Abstract

Fundamental insight into excimer formation has been gained by using 9,10-bis[4-(9-carbazolyl)phenyl]anthracene] (**Cz^9^PhAn**) as a probe. **Cz^9^PhAn** exhibits a highly emissive blue fluorescence in solution and is found to emit a panchromatic white light spectrum (400–750 nm) in film, powder and single crystal, in which an additional excimer band appears at ∼550 nm. Detailed structural analyses, emission relaxation dynamics and a theoretical approach conclude the formation of an anthracene*/phenyl ring excimer through an overlap between π* (anthracene) and π (phenyl ring) orbitals in a face-to-edge stacking orientation. The rate of excimer formation is determined to be 2.2 × 10^9^ s^–1^ at room temperature, which requires coupling with lattice motion with an activation energy of 0.44 kcal mol^–1^. Exploiting **Cz^9^PhAn** as a single emitter, a fluorescent white organic light emitting diode (WOLED) is fabricated with a maximum external quantum efficiency (*η*_ext_) of 3.6% at 1000 cd m^–2^ (4.2 V) and Commission Internationale de L'Eclairage (CIE) coordinates of (0.30, 0.33). The white-light **Cz^9^PhAn** reveals a preferred orientation of the transition dipole moment in the emitting layer to enhance light outcoupling. This non-doped, single component (**Cz^9^PhAn**) WOLED greatly reduces the complexity of the fabrication process, rendering a green and cost-effective alternative among the contemporary display/lighting technologies.

## Abstract 2

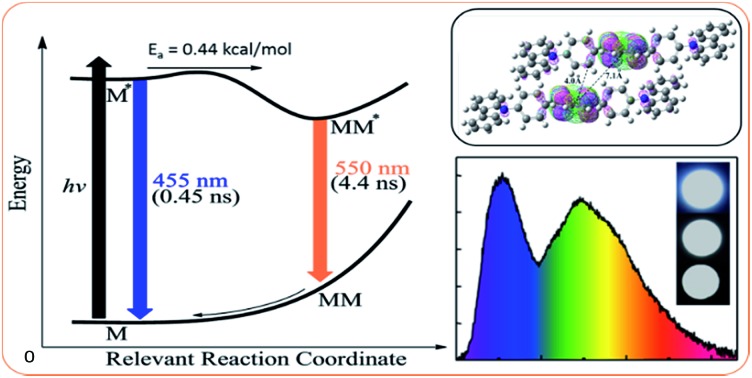
Fundamental insight into excimer formation of **Cz^9^PhAn**, achieving a single-component, high-performance WOLED.

## 1. Introduction

White organic light-emitting diodes (WOLEDs) have attracted broad attention for potential applications in flat-panel displays and solid-state lighting.^1^ WOLEDs can be engineered with single or multiple active layers to attain white light emission. Multiple active layer designs normally incorporate different chromophores into each active layer, while multiple chromophores are commonly required and mixed in the single active layer design. Sun *et al.* reported a WOLED device using a multiple active-layer structure with a blue fluorescent dopant and phosphorescent dopants (for green and red). This device exhibited high internal efficiency (∼100%) with a color rendering index (CRI) of 85 and Commission Internationale de L'Eclairage (CIE) coordinates of (0.40, 0.41).^2^ More recently, Zhou *et al.* have reported a device based on the blue fluorescence of 4,4′-bis-[*N*-(1-naphthyl)-*N*-phenylamino]biphenyl (NPB), with the green and red phosphorescence of a platinum complex to duplicate sunlight, exhibiting an extraordinarily high CRI of 97.^3^ Wong and co-workers reported a bipolar universal host, *o*OXA, combining RGB phosphors within a single emitting layer to realize WOLEDs with external quantum efficiency of 20%, high CRI of 87 and stable chromaticity.^4^

In addition to the mixing of RGB colors, white light can in fact be simply generated by ratiometric fine-tuning of two complementary colors of light, for example, blue and yellow, on the CIE color space. Park and co-workers reported a WOLED comprising the covalently linked blue and yellow color-emitting fluorophores of proton transfer molecules.^5^,^6^ Chou *et al.* reported a pure white-light emitting mechanism developed by anchoring organic yellow-light-emitting fluorophores onto inorganic ultra-small CdSe quantum dots (blue-light emitting).^7^ However, both approaches, the use of RGB and two complementary colors, require a delicate balance for both excitation and emission between mixed multiple materials. In particular, color balancing becomes much more complicated when the inter-chromophore energy transfer mechanism is involved. These, together with the various aging lifespans of each emissive material, which may cause spectral variation collaterally, would certainly hamper any practical application.

Thus, the development of a single emitting material capable of emitting white light, preferably panchromatic white light, is demanding. As for harvesting phosphorescent white light, a single-doped WOLED with 20% external efficiency has been achieved;8*b* it is based on a phosphorescent Pt complex in a relatively complicated device structure: a co-host of TAPC : PO15 (1 : 1) for a 10%-doped Pt device. White light was generated by coupling blue-green phosphorescence from single Pt complexes and orange emission from Pt aggregates. However, phosphorescent OLEDs (PHOLEDs) are hampered by the high cost and rarity of the noble metal elements. Alternatively, WOLEDs employing metal-free single-emitters have emerged as cheaper substitutes for PHOLEDs and there has been rapid development of these into single-emitter fluorescent WOLEDs by various methods such as pH-dependent emission,9*a* thermo-responsive emission9*b* and intermolecular charge transfer (ICT).9*c*

In light of pursuing single-molecule-active-layered fluorescent WOLEDs, we recently reported a single-molecule white light-emitting system, 1-hydroxy-11*H*-benzo[*b*]fluoren-11-one,^10^ based on its reversible excited-state intramolecular proton transfer reaction, such that both normal (blue) and proton-transfer tautomer (yellow) emissions appear to give white light with CIE coordinates at (0.30, 0.27). Among other WOLEDs fabricated from various single-component materials,^11^–^16^ carbazole derivative-based devices have been demonstrated to have great color purity.^11^–^13^ For instance, Hou and coworkers developed two carbazole-substituted aromatic enynes as single-white light emitting materials in a double-layer device structure.^12^ Moreover, anthracene-cored compounds were utilized not only for blue emitting derivatives but also for white emitting frameworks due to their excellent high photoluminescence (PL) quantum efficiency and good thermal stability.^14^–^19^ Zhang and coworkers synthesized a triphenylamine/pyrenylanthracene-composed material, DPAA, which was fabricated as the emitting layer in a three-layer device exhibiting white light emission. The visible light emission spectra of these materials were commonly derived from normal (monomer) and excimer fluorescence bands. Our recent advance revealed that the color purity and the efficiency of the OLED device can be fine-tuned by modifying the position of the carbazole moiety on the anthracene derivative and/or the π-spacer conjugation length between the carbazole moiety and anthracene.^20^,^21^ Although these carbazole-substituted anthracene derivatives exhibited only blue emission suitable for non-doped blue devices, it is feasible, on the basis of these in-depth findings, to tailor and fine-tune the molecular structure in an aim to attain panchromatic light with high quantum efficiencies.

**Scheme 1:**
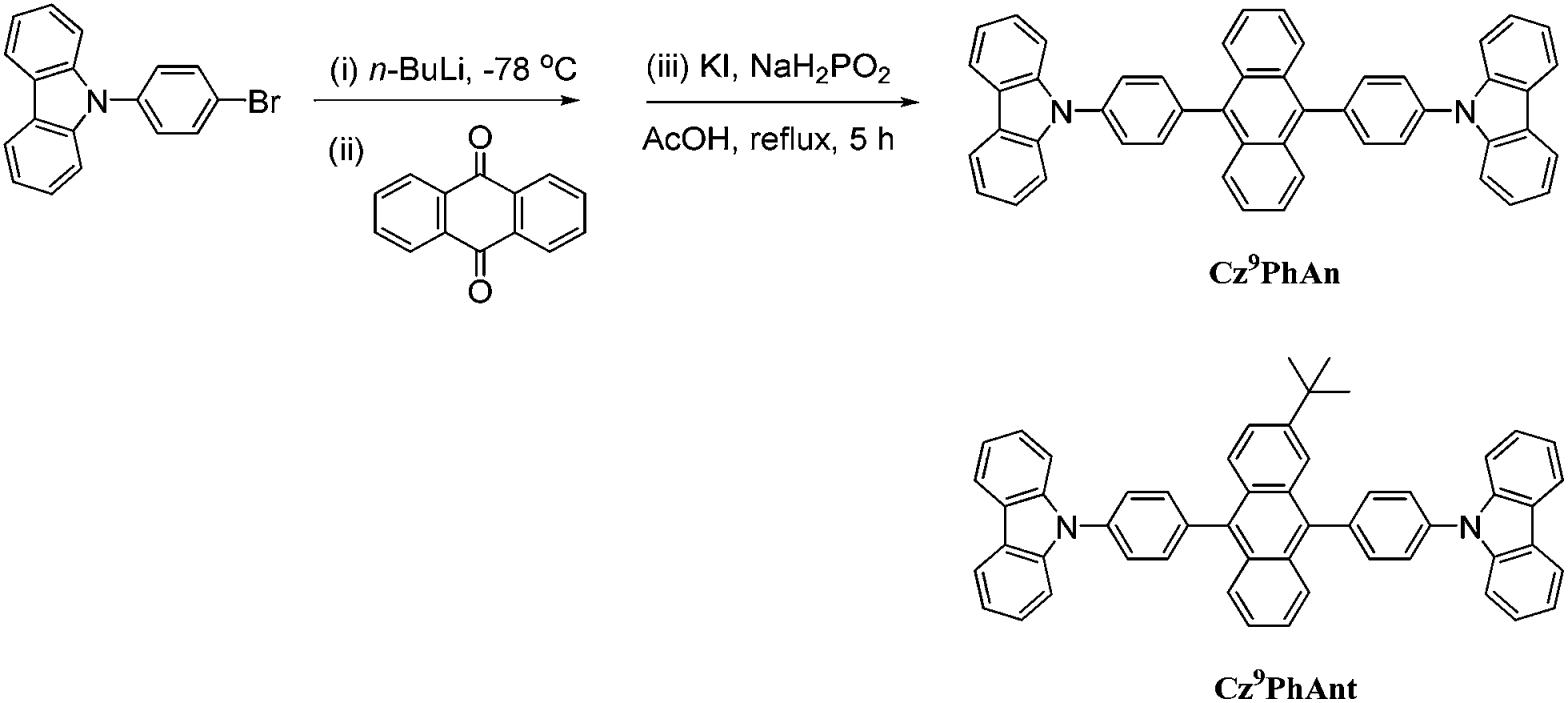
Synthetic routes toward **Cz^9^PhAn**. Also shown is the structure of a **Cz^9^PhAn** analogue, **Cz^9^PhAnt**, for comparison.

We report herein the synthesis (see ESI†), characterization, and WOLED application of a new material, **Cz^9^PhAn** (Scheme 1), comprising two 9-phenylcarbazole units covalently linked to the C9 and C10 positions of anthracene. **Cz^9^PhAn** exhibits prominent excimer emission in single crystal and solid film. While excimer generation may not be uncommon in lighting devices, the fundamental insight into the mechanism of excimer-associated white light generation is still pending. The goal of this study is thus to gain understanding of the fundamentals of excimer formation *via* structural analyses and the associated spectroscopy and reaction dynamics. Furthermore, the combination of dual, far-separated and high-intensity monomer (450 nm) and excimer (540 nm) emissions in **Cz^9^PhAn** permits remarkable white light generation. As a result, a non-doped WOLED incorporating **Cz^9^PhAn** as the emitter is successfully fabricated, showing a high value of *η*_ext_ of 3.6% at 1000 cd m^–2^ with CIE coordinates of (0.30, 0.33), which should be more attractive than doped WOLEDs because the fabrication processes are facile and the devices are more reliable. Details of results and discussion are elaborated as follows.

## 2. Results and discussion

**Fig. 1:**
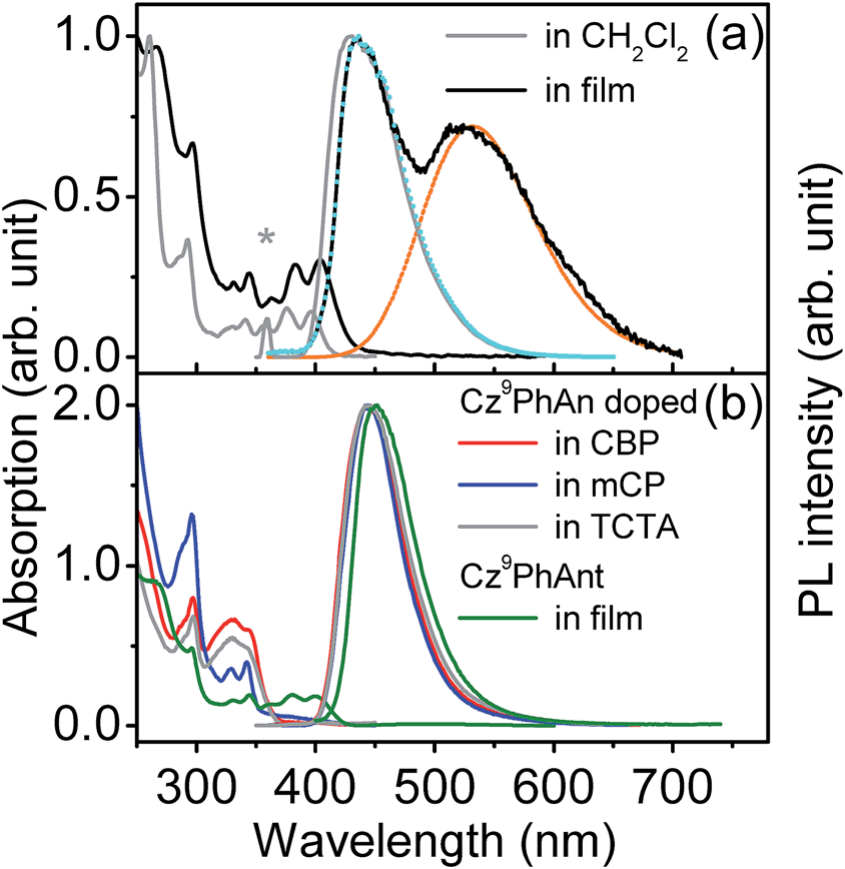
(a) Steady-state absorption and photoluminescence spectra of **Cz^9^PhAn** in CH_2_Cl_2_ and film (prepared *via* thermal evaporation). Blue and orange dashed lines represent the spectral decomposition of the film photoluminescence spectrum (see text for assignments and details). The asterisk shows the excitation wavelength. (b) Steady-state spectra of **Cz^9^PhAnt** film and 3 wt% **Cz^9^PhAn** doped in a common host (prepared *via* thermal evaporation).

**Fig. 2:**
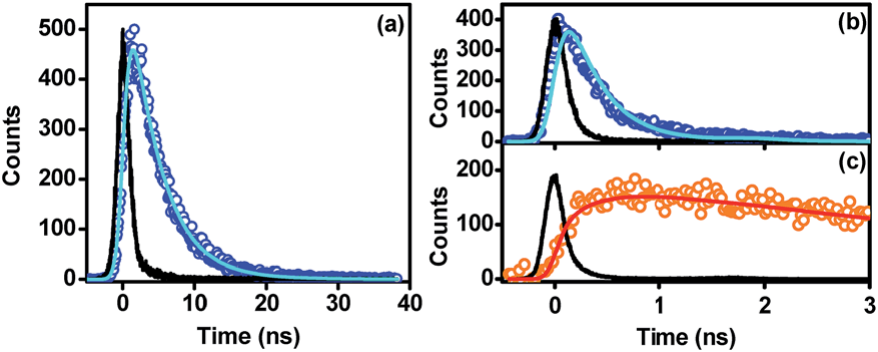
Time-resolved photoluminescence decay curves of **Cz^9^PhAn** in dichloromethane solution monitored at 430 nm (a), and **Cz^9^PhAn** film sample monitored at 440 nm (b) and 600 nm (c). Open circles denote TCSPC data points, while solid lines denote the poly-exponential fitting of the data, except for the black lines, which represent instrument response functions. The excitation wavelength is 380 nm. Fitting results are summarized in Table 1.

Fig. 1a shows the steady-state absorption and emission spectra of **Cz^9^PhAn** dissolved in dichloromethane. Pertinent photophysical properties are summarized in Table 1. The lowest lying absorption band is at *λ*_max_ ∼ 396 nm with vibronic structures, and the emission band peaks at 430 nm. The absorption and emission spectra show a good mirror-image relationship with a common Stokes shift (difference in frequency between lowest-lying absorption peak and emission peak) of ∼2000 cm^–1^. The measured emission (fluorescence) quantum yield of **Cz^9^PhAn** in dichloromethane is near unity (∼1.0). Further time-resolved fluorescence measurements (see Fig. 2a) revealed a single exponential fluorescence decay with a time constant of 4.1 ns (*χ*^2^ = 1.02). Accordingly, the radiative decay rate constant was then calculated to be as large as 2.4 × 10^8^ s^–1^, implying that the lowest lying absorption is a fully allowed π–π* transition. The absorption and emission spectra of a **Cz^9^PhAn** film sample (prepared by thermal evaporation) are also shown in Fig. 1a. Both the absorption (peak at 404 nm) and normal emission (peak at 445 nm) spectra are slightly red-shifted with respect to those in dichloromethane solution. Remarkably, however, an additional emission band appears with a peak wavelength at 550 nm. The excitation scan, monitored at either 445 nm or 550 nm, shows an identical excitation spectral profile, which also resembles the absorption spectrum, indicating that both the 445 nm and 550 nm emission bands originate from a common ground state. Accordingly, the assignment of this 550 nm band in the **Cz^9^PhAn** solid film to an excimer emission is reasonable.

**Table 1:** Photophysical Properties of **Cz^9^PhAn**

**Cz^9^PhAn**	Solution (CH_2_Cl_2_)	Neat film	Doped in film of CBP	Doped in film of mCP	Doped in film of TCTA
*λ*_Abs_ (nm)	396	404	404	404	404
*λ*_Em_ (nm)	430	445; 550	445	445	445
*φ* Q.Y.	∼1	0.25; 0.22	∼1	∼1	∼1
*τ*_Em_ ^*a*^ (ns)	4.1	0.45; 4.4^*c*^	3.9	4.5	4.2
*k*_r_ ^*b*^ (s^–1^)	2.4 × 10^8^	5.6 × 10^8^; 6.7 × 10^7^^*d*^	2.6 × 10^8^	2.2 × 10^8^	2.4 × 10^8^

^*a*^ *τ*_Em_: fitted emission lifetime.

^*b*^ *k*_r_: calculated radiative decay rate constant.

^*c*^Accompanied with an initial fast rise component whose time constant is fitted to 0.47 ns (470 ps).

^*d*^A lower limit calculated with the assumption of the upper limit of branching (0.75) of excimer formation.

Further support of this viewpoint is given by the dilution of the **Cz^9^PhAn** solid film. Upon doping of **Cz^9^PhAn** onto a host material such as 4,4′-bis(*N*-carbazolyl)-1,1′-biphenyl (CBP), 1,3-bis(*N*-carbazolyl)benzene (mCP) and 4,4′,4′′-tri(*N*-carbazolyl)triphenylamine (TCTA), forming a diluted **Cz^9^PhAn** solid film (3% by weight), only 445 nm emission appears, and no 550 nm emission band (see Fig. 1b and Table 1). Perhaps the firmest support of excimer formation is given by the time-resolved emission analyses of a **Cz^9^PhAn** film sample shown in Fig. 2b and c. Time-correlated single photon counting (TCSPC) measurements monitored at 440 nm (Fig. 2b) show a short emission decay time of 0.45 ns (450 ± 30 ps), which is in sharp contrast (∼10 times faster) to the fluorescence decay of **Cz^9^PhAn** in CH_2_Cl_2_ (Fig. 2a) as well as **Cz^9^PhAn** doped (3% by weight) in CBP, mCP and TCTA films (see Table 1 and Fig. S1 in the ESI†). On the other hand, as shown in Fig. 2c, the time-resolved emission decay monitored at 600 nm clearly reveals a rise and a decay component, whose time constants were fitted to be 470 ± 30 ps and 4.4 ns, respectively. Notably, within experimental error, the rise time of the excimer emission band matches well with the decay time of the normal emission band. The associated kinetics can thus be well described by a precursor–successor type of relationship between monomer (precursor) and excimer (successor) emission. With the confirmation of the excimer formation and subtraction of the normal (monomer) emission profile obtained from the diluted film spectra (Fig. 1b), we were able to spectrally decompose the film spectrum into normal (monomer) and excimer photoluminescence and hence determine their corresponding emission quantum yields (see blue and orange curves in Fig. 1a).

**Fig. 3:**
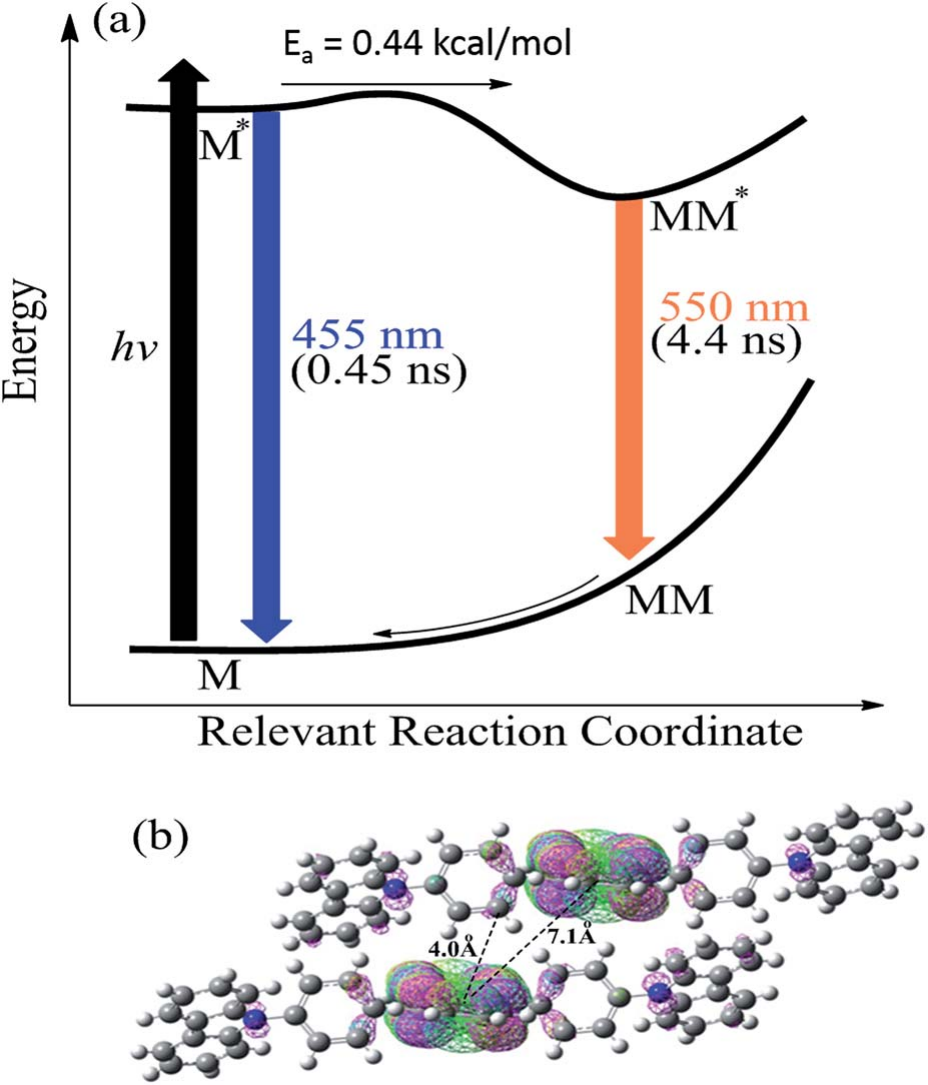
(a) A schematic diagram illustrating the photodynamics of **Cz^9^PhAn** solid thin film upon photoexcitation. *E*_a_ 0.44 (kcal mol^–1^): the activation energy of excimer formation. (b) The side view of two **Cz^9^PhAn** slabs for which excimer formation may be associated with M–M* (M denotes **Cz^9^PhAn**) interaction assisted by the lattice motion. Also shown are the face-to-edge frontier orbitals of two **Cz^9^PhAn** slabs. HOMO is in pink and LUMO is in green.

**Fig. 4:**
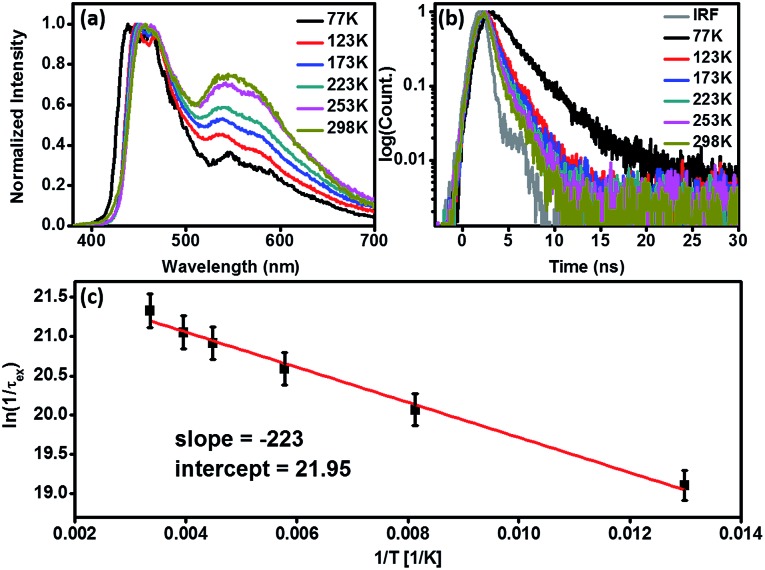
Temperature-dependent properties of neat **Cz^9^PhAn** film (prepared *via* thermal evaporation). (a) Photoluminescence spectra, note: the emission is normalized at the maximum of the monomer emission (445 nm) for comparison. (b) Time-resolved photoluminescence decay curves monitored at 440 nm. (c) Arrhenius plot of the rate constant (l/*τ*_ex_) *versus* reciprocal temperature.

Fig. 3 schematically summarizes the excited state dynamics and the emission of both monomeric and excimeric excitons in the solid state upon photoexcitation. In diluted CH_2_Cl_2_ solution and **Cz^9^PhAn** (3wt%) doped solid film, the lifetime of **Cz^9^PhAn** in the excited state is much shorter than its diffusion time, explaining the absence of excimer emission. As for the solid **Cz^9^PhAn**, excimer formation *via* exciton-diffusion is feasible for the molecules that are in close contact with each other due to tight molecular packing. Temperature-dependent photoluminescence and the associated relaxation dynamics were then studied to gain the detailed kinetic parameters of the excimer formation. Upon lowering the temperature from 298–77 K in neat **Cz^9^PhAn** film, as shown in (Fig. 4), the results clearly show that the excimer 550 nm emission band gradually decreases, accompanied by an increase in the monomer 440 nm emission. Correspondingly, the decay rate constant *k*_neat_(*T*) of the monomer emission increases from 0.45 (298 K) to 2.2 ns (77 K), supporting the thermally activated excimer formation. We also measured the temperature-dependent relaxation dynamics of the 3wt% **Cz^9^PhAn** doped film, *i.e.*, *k*_dilute_(*T*), where only normal emission is resolved. The temperature-dependent rate constant of the excimer formation *k*_ex_(*T*) can thus be deduced using eqn (1) and an Arrhenius plot for ln *k*_ex_(*T*) as a function of 1/*T* can be expressed in eqn (2), where *E*_a_ and *A* denote the reaction activation energy and frequency factor, respectively.

1 *k*_ex_(*T*) = *k*_neat_(*T*) – *k*_dilute_(*T*)

2
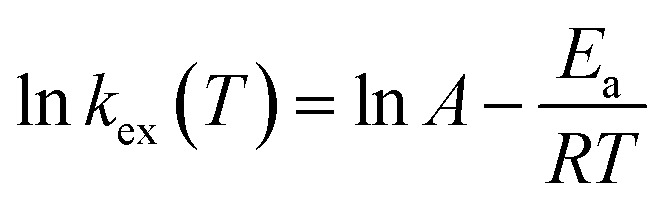



The results shown in Fig. 4c reveal a straight line and consequently, *E*_a_ and *A* for the excimer formation process are calculated to be 0.44 kcal mol^–1^ and 3.41 × 10^9^ s^–1^, respectively. The small frequency factor (∼30 cm^–1^) indicates that the low frequency motion, most likely the motion associated with lattice vibration, would induce the excimer formation.

**Fig. 5:**
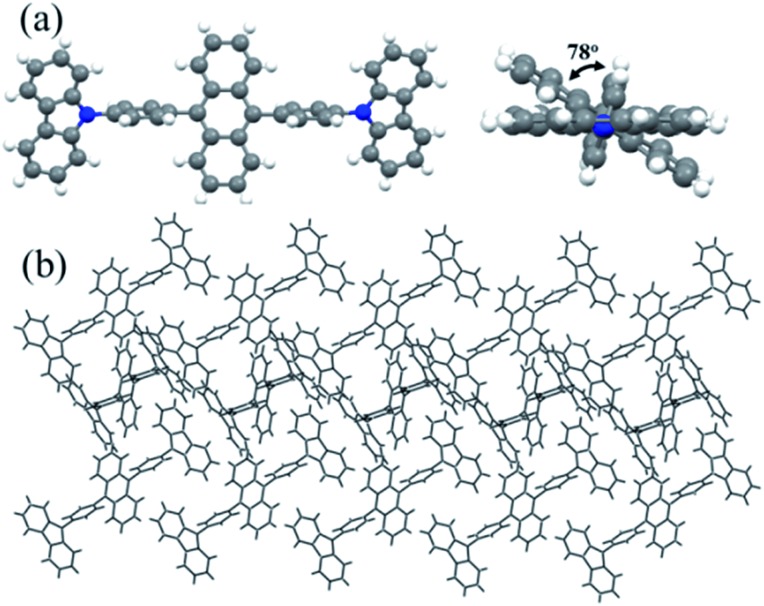
X-ray crystallographic analysis of **Cz^9^PhAn**. (a) Ball-and-stick models of **Cz^9^PhAn** viewed perpendicular to the anthracene plane (left) and along the carbazole plane (right). (b) Crystal packing diagram of **Cz^9^PhAn**.

As shown in Fig. S2 of the ESI,† **Cz^9^PhAn** in a single crystal also exhibits dual emission maximized at 450 and 560 nm. Therefore, X-ray structural analyses may render a clue regarding the excimer formation. Notably, single crystal X-ray diffraction analysis, shown in Fig. 5 and S3,† indicated that two-dimensional intermolecular networks are formed by the face-to-edge stacking geometries in the crystal, and the step-like formation of **Cz^9^PhAn** was established unequivocally. Meanwhile, the peripheral carbazole moiety will also act as an intermolecular contact platform, adding further stabilization *via* the weak π-stacking interactions.^22^ These types of arrangement may be beneficial for the excimer formation in the solid state.^23^ As a comparison, the addition of a *tert*-butyl group on the anthracene moiety of **Cz^9^PhAn** forms **Cz^9^PhAnt** (see Scheme 1). **Cz^9^PhAnt** has been demonstrated as a blue emitter,^20^ exhibiting a normal emission (450 nm) only in solid film (see Fig. 1b). From the viewpoint of the chemical structure of **Cz^9^PhAnt**, the steric effect of *tert*-butyl groups has proved to be an efficient methodology for reducing the intermolecular interaction and suppressing excimer emission.^24^ As a result, relatively loose molecular packing was resolved for **Cz^9^PhAnt**, as indicated by the lack of excimer emission spectra. Obviously, removing the *tert*-butyl group at the 2-position of anthracene increases the strength of the intermolecular interaction between adjacent **Cz^9^PhAn** molecules, leading to good overlap of the vibronic wavefunction in the electronically excited state23*b**via* the assistance of, *e.g.*, lattice motion (*vide supra*).

The time-dependent DFT (B3LYP/6-31G(d,p)) calculations of **Cz^9^PhAn** showed the lowest lying transition to be the highest occupied molecular orbital (HOMO) → the lowest occupied molecular orbital (LUMO) in character, for which the HOMO and LUMO are mainly located at the anthracene moiety in both the monomer or the dimer-like structure truncated from the X-ray crystal structure of **Cz^9^PhAn** (see Fig. S4†). Nevertheless, a small but non-negligible portion of the HOMO is located at the bridging phenyl ring. Careful examination of the X-ray structure of **Cz^9^PhAn** also indicates that the nearest anthracene (center) to anthracene (center) distance between two adjacent slabs is as far as ∼7.1 Å (see Fig. 3b). Thus, the possibility of excimer formation originating from the anthracene*/anthracene (* denotes the electronically excited state) pair is discarded. Alternatively, we noticed that the nearest distance between the phenyl bridge of one slab and center of anthracene of the adjacent slab is as short as 4.0 Å (see Fig. 3b) while they are mutually twisted with a dihedral angle of 78° (see Fig. 5). Additionally, the top view of the two slabs clearly shows that the center of anthracene is closely in line with the bridging phenyl plane (see Fig. S3†). The calculation shown in Fig. 3b also indicates the potential approach of frontier orbitals between the anthracine of one slab and the phenyl bridging ring of the adjacent slab. We thus tentatively propose an anthracene*/phenyl ring excimer-like formation where the interaction is through an overlap between π* (anthracene) and π (phenyl ring) orbitals in a face-to-edge-like orientation, the energy of which is then minimized by coupling the internal lattice motion that determines the rate of excimer formation. Note that in this case, the term “excimer” holds because the process still incorporates two **Cz^9^PhAn** molecules in the excited and ground states, respectively. We realize that this type of face-to-edge excimer formation, though unusual, is plausible from the theoretical point of view.^25^

We then performed computation by utilizing the two truncated slabs of **Cz^9^PhAn** (see Fig. 3b) to gain further insight into the formation mechanism. Attempts by full geometry optimization (DFT at the B3LYP/6-31G(d,p) level and TD-DFT) in the excited state unfortunately failed, perhaps due to a rather small interaction between these two slabs. Alternatively, we then gradually decreased the parallel distance between two anthracene planes within the slabs, starting from 5.10 Å (the X-ray data), by a decrement of 0.01 Å. According to the repulsion (attraction) of the potential energy surface in the ground (excited) states (see Fig. 3a), we may anticipate a smooth change or perhaps a (local) minimum of the S_0_ → S_1_ transition around the excimer formation region. As a result, at a parallel distance of 4.03 Å between two slabs, a transition of 430 nm (23 256 cm^–1^, see Fig. S5†) was calculated, for which the adjacent transition (with 0.01 Å resolution) is higher in transition energy. This value is close to the onset of the excimer emission at ∼450 nm (see the fitting curve in Fig. 1a). This approach, though being qualitative, supports the feasibility of excimer formation *via* certain lattice motions to shorten the inter-slab distance.

**Fig. 6:**
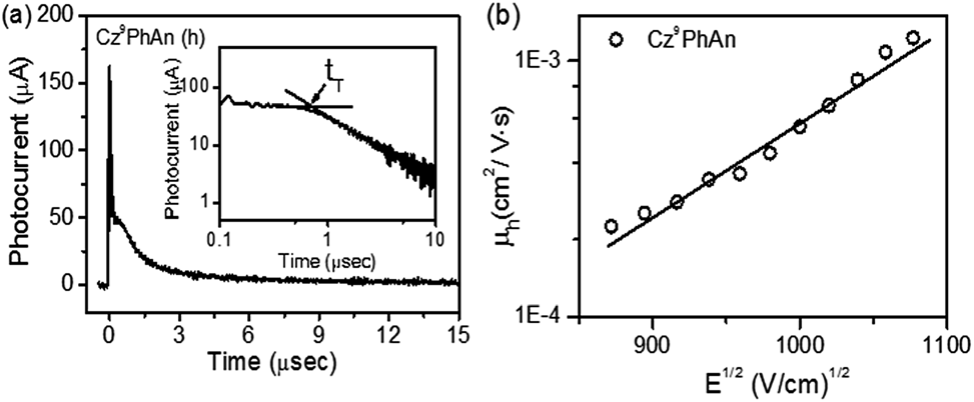
(a) Representative TOF transients for **Cz^9^PhAn** (thickness: 1.55 μm; *E* = 4.5 × 10^5^ V cm^–1^). Inset: double logarithmic plot. (b) Hole mobility of **Cz^9^PhAn** plotted with respect to *E*^1/2^.

Experimentally, the HOMO levels were determined using ultraviolet photoelectron spectroscopy. The LUMO energy level was estimated from the HOMO level and the optical band gap. The corresponding HOMO/LUMO energy levels of **Cz^9^PhAn** were calculated to be –5.83/–2.95 eV. To further understand the charge-carrier transport properties, we used the time-of-fight (TOF) technique to evaluate the carrier mobilities. Representative TOF transients for holes of **Cz^9^PhAn** are revealed in Fig. 6a. Fig. 6b shows the hole mobility plotted as a function of the square root of the electric field. The hole mobility of **Cz^9^PhAn** lies in the range from 4.9 × 10^–4^ to 4.5 × 10^–3^ cm^2^ V^–1^ s^–1^ for fields varying from 4.5 × 10^5^ to 7.9 × 10^5^ V cm^–1^. In view of the dual emission (monomer and excimer) of **Cz^9^PhAn**, the application of this material as an emitter for a white OLED was investigated using a simple configuration: indium tin oxide (ITO)/HAT-CN (10 nm)/NPB (60 nm)/TCTA (5 nm)/**Cz^9^PhAn** (25 nm)/TPBI (50 nm)/LiF (0.5 nm)/Al (100 nm). To improve the hole injection from the anode, we used 4,5,8,9,11-hexaazatriphenylene-hexacarbonitrile (HAT-CN)^26^ as a hole injection layer. Two hole-transporting layers (HTLs), which consisted of a 20 nm-thick layer of 4,4′-bis[*N*-(1-naphthyl)-*N*-phenyl]biphenyldiamine (NPB)^27^ and a 5 nm-thick thick layer of 4,4′,4′′-tri(*N*-carbazolyl)triphenylamine (TCTA),^28^ were implemented. It is noteworthy that TCTA (HOMO level of –5.7 eV) was inserted between NPB and **Cz^9^PhAn** with a stepwise increase in HOMOs into the emitting layer. To further confine the holes or the excitons generated within the emissive region, 1,3,5-tris(*N*-phenylbenzimidizol-2-yl)benzene (TPBI)^29^ with a high-energy gap was selected as the electron-transporting layer (ETL). LiF and Al served as an electron-injecting layer and cathode, respectively.

**Fig. 7:**
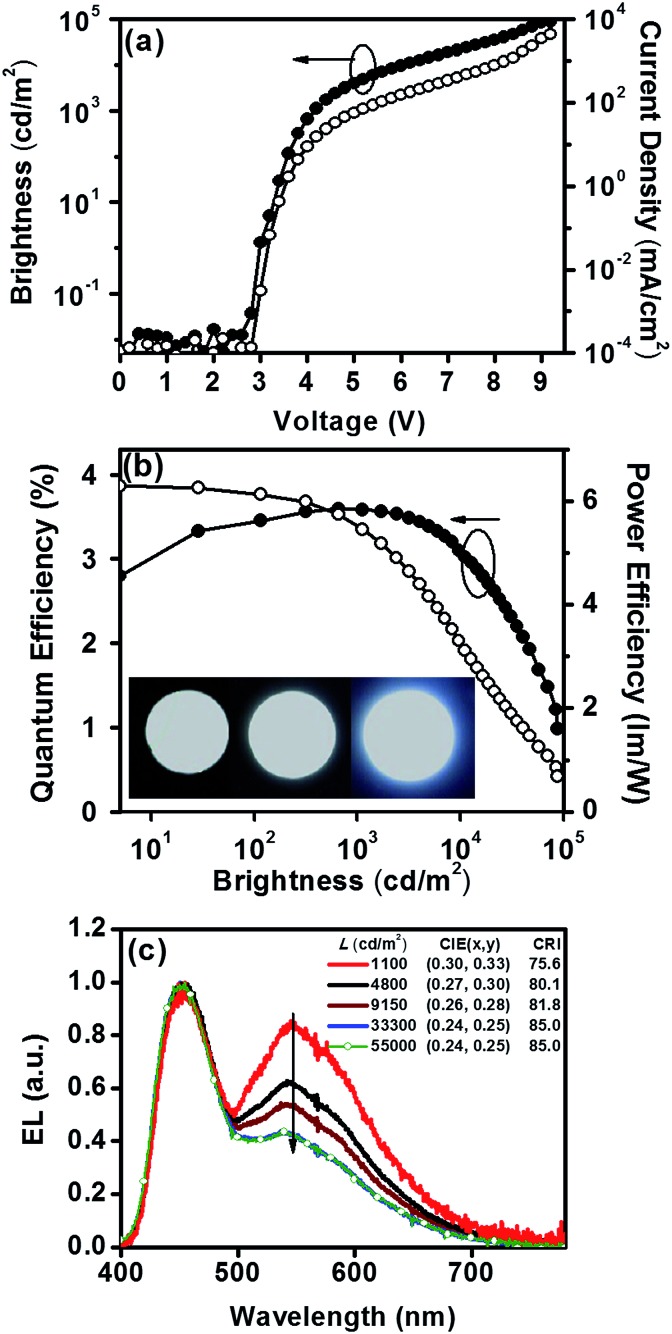
(a) Current density–voltage–luminance (*J*–*V*–*L*) characteristics, (b) external quantum (*η*_ext_) and power efficiencies (*η*_P_) as a function of brightness, and (c) normalized EL spectra of the white device.

**Fig. 8:**
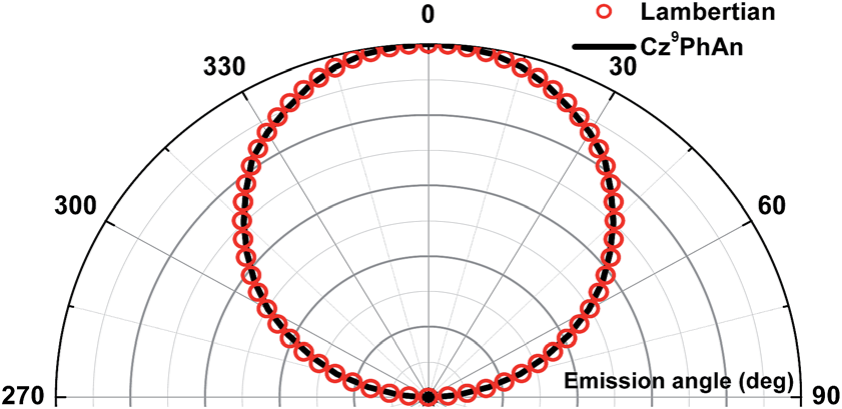
Angular distribution of radiation irradiance for the white device and the Lambertian emission pattern (normalized to the 0° irradiance).

Devices employing **Cz^9^PhAn** as an emitter are compared in Fig. 7. The **Cz^9^PhAn** device displayed a turn-on voltage of 2.8 V, and the driving voltage of the WOLED at 1000 cd m^–2^ was about 4.2 V (15 mA cm^–2^), achieving a maximum external quantum efficiency (*η*_ext_) of 3.6%, corresponding to a current efficiency (*η*_c_) of 8.5 cd A^–1^ and power efficiency (*η*_p_) of 6.3 lm W^–1^. The EL emission spectra had two distinct emission bands, a blue band (monomer) centered at 453 nm and a broad yellow band (excimer) with a peak at *ca.* 545 nm, and demonstrated white emission with a high color-rendering index (CRI = 75.6–85.0), as shown in Fig. 7c. The intensity ratio for the excimer *versus* normal emission decreased with increasing brightness from 1100 to 33 300 cd m^–2^, and then remained unchanged up to 55 000 cd m^–2^ (see Fig. 7c) and even higher, leading to a slight shift of the CIE coordinates from (0.30, 0.33) to (0.24, 0.25). The driving-voltage-dependent emission ratio is commonly interpreted by mismatch between the electron and hole transfer rates in the active layer, resulting in an uneven exciton distribution. However, this normally occurs in the host–dopant system,^8^ which may not be operative for the non-dopant system in this study. Alternatively, because the excimer is in the lower energy level, the cascade from the blue (monomer) emitter to the yellow (excimer) might account for the ratiometric change of dual emission. Under lower current (low brightness), the emission ratio is driving-voltage-dependent, while it remains unchanged upon reaching saturation at high current (high brightness). Remarkably, the device showed rather low efficiency roll-off at a brightness of 1000 cd m^–2^, while the recorded *η*_ext_ remained as high as 3.6% with CIE coordinates of (0.30, 0.33). Angle-dependent EL intensity measurements show the EL intensity distributions of the device as well as the ideal Lambertian distribution for comparison (Fig. 8).

**Fig. 9:**
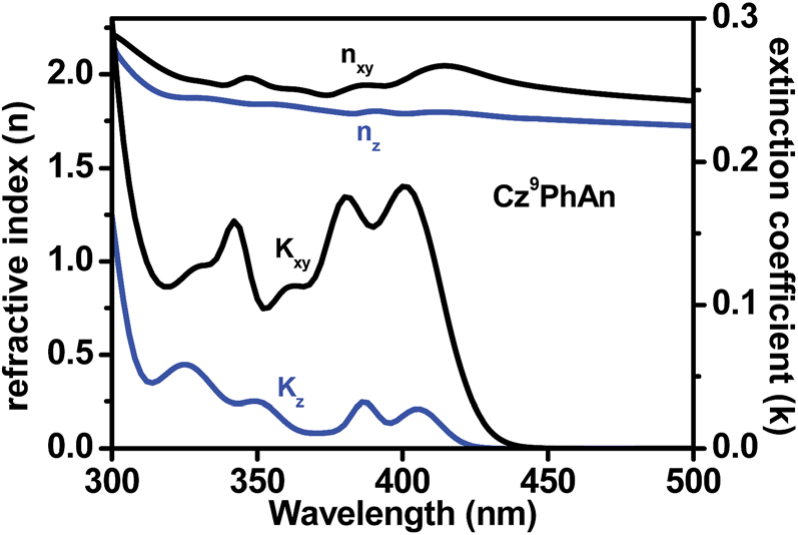
Optical anisotropy of the **Cz^9^PhAn** film: anisotropic refractive indices *n* and extinction coefficients *k*. The black lines (*n*_*xy*_ and *k*_*xy*_) and blue lines (*n*_*z*_ and *k*_*z*_) indicate the in-plane (horizontal) and out-of-plane (vertical) components of the optical constants, respectively.

Considering the PLQY of 0.47, a singlet ratio around 25%, and a usual optical out-coupling efficiency of 20% for typical OLEDs, one would only expect an upper limit of *ca.* 2.4% in *η*_ext_ with such a PLQY. Careful analysis indicates that this superior efficiency is attributed to the preferred horizontal orientation of the transition (emitting) dipoles that would significantly enhance the light outcoupling efficiency (*η*_out_) of the OLED.^30^Fig. 9 shows the anisotropies in the refractive index and extinction coefficient as a function of wavelength. The extinction coefficient of the out-of-plane (vertical) component (blue line) is small compared to that of the in-plane (horizontal) component (black line). This result indicates that the transition dipole moments of **Cz^9^PhAn** are mainly oriented horizontally, leading to a high optical out-coupling effect when emitting light from such dipoles. According to previous studies,^30^ highly horizontally oriented emitting dipoles could result in an up to 2-fold out-coupling enhancement, compared to isotropic emitters.

**Fig. 10:**
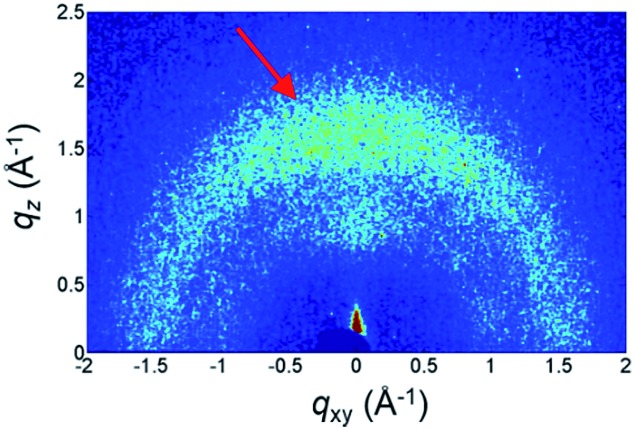
GIWAXS pattern of **Cz^9^PhAn** thin film. The arrow indicates the diffraction ring of the face-to-edge stacking.

To gain further insight, the Grazing Incidence Wide Angle X-ray Scattering (GIWAXS) pattern of the **Cz^9^PhAn** thin film is shown in Fig. 10. A broad diffraction ring is seen for the film, as indicated by the red arrow. The corresponding *d*-spacing is 4.0 Å for **Cz^9^PhAn**, which can be rationally assigned to the periodical face-to-edge stacking of the anthracene and phenyl ring. Furthermore, the film shows an uneven diffraction ring with stronger intensity at the *q*_*z*_ axis, implying a preferential stacking of the molecules along the thickness direction of the film, which evidences the horizontal orientation of the transition dipoles that enhances the light outcoupling efficiency.

The facile synthetic scheme with high product yield (>70%) makes it feasible to scale-up the synthesis of **Cz^9^PhAn** to hundreds of grams in the lab. Using this approach, we then further fabricated several prototype lighting devices with active illuminating areas of either 5 × 10 cm^2^ or 5 × 5 cm^2^. The resulting WOLEDs, shown in Fig. S6 of the ESI,† demonstrate the great potential of this single-molecule-active-layered, panchromatic WOLED for pragmatic application.

## 3. Conclusions

To sum up, a new single white light-emitting material, **Cz^9^PhAn**, was designed and synthesized. The tight intermolecular packing, resulting from the face-to-edge interactions between the phenyl bridge of one slab and the center of anthracene of the adjacent slab, is capable of producing excimer emission. Consequently, the panchromatic emission of **Cz^9^PhAn** is contributed by its normal (blue) and excimer (yellow) fluorescence, as supported by the combination of X-ray analyses and the corresponding photophysical studies. A single-component WOLED incorporated with **Cz^9^PhAn** as an emitter exhibits a high value of *η*_ext_ of 3.6% at 1000 cd m^–2^ (4.2 V) with CIE coordinates of (0.30, 0.33). The **Cz^9^PhAn** film revealed a preferred orientation of the transition dipole moment in the emitting layer to enhance light outcoupling. Knowing that the excimer formation commonly originates from π-stacking of the framework in the film, the enhanced light outcoupling in the device is expected, which is exactly what we observed in this study. The non-doped **Cz^9^PhAn** WOLED demonstrates that fluorescent white light generation can be achieved in a single-molecule system with pragmatic WOLED performance, which is certainly competitive with other contemporary display technologies in terms of simplicity and hence cost-effectiveness and reliability. Our comprehensive structural and spectroscopic/dynamics study of the excimer emission and the record high efficiency of the single-molecule type fluorescent WOLED are to be far-reaching in the future due to the in-depth understanding of mechanism acquired in this study.

## Supplementary Material

Supplementary information

Crystal structure data
