# Supplementary material for: Insight into the mechanism and outcoupling enhancement of excimer-associated white light generation
Source: Chem Sci. 2016 Feb 12;7(6):3556–63. doi: 10.1039/c5sc04902d (PMC6007176; doi:10.1039/c5sc04902d)
Supplement: Supplementary file 1 [file SC-007-C5SC04902D-s001.pdf]

Electronic Supplementary Material (ESI) for Chemical Science.
This journal is © The Royal Society of Chemistry 2016

Supporting Information

Insight into the mechanism and outcoupling enhancement of excimer-associated white light generation

Ying-Hsiao Chen,[a†] Kuo-Chun Tang,[a†] Yi-Ting Chen,[a] Jiun-Yi Shen,[a] Yu-Sin Wu,[a] Shih-Hung Liu,[a] Chun-Shu Lee,[b] Chang-Hsuan Chen,[b] Tzu-Yu Lai,[c] Shih-Huang Tung,[c] Ru-Jong Jeng,*[c] Wen-Yi Hung,*[b] Min Jiao,[d] Chung-Chih Wu[d] and Pi-Tai Chou*[a]

Contents

	Page
Experimental Section	S2
**Figure S1**. Time-resolved photoluminescence decay curves of a solid film in which **Cz^{9}PhAn** is doped in a host material (CBP, mCP and TCTA, see text for detail)	S6
**Figure S2.** The emission spectrum of **Cz^{9}PhAn** in a single crystal	S6
**Figure S3.** Molecular packing of **Cz^{9}PhAn** from a top view	S7
**Figure S4.** HOMO and LUMO frontier orbitals distribution for **Cz^{9}PhAn** calculated using DFT methods (B3LYP 6-31G*)	S7
**Figure S5**. The side view of two truncated **Cz^{9}PhAn** slabs. $S_0 \rightarrow S_1$ at 430 nm, f = 0.1422, HOMO (pink mesh) → LUMO (green mesh) (98%). The distance between anthracenes is calculated to be 4.03 Å	S10
**Figure S6.** A prototype demonstration of our single molecule type WOLED lighting device based on **Cz^{9}PhAn** with the size of 5 × 10 cm^2 or 5 × 5 cm^2	S10
**Figure S7.** (a) TGA and (b) DSC spectra of **Cz^{9}PhAn**	S10
**Table S1.** Crystal data and structure refinement details for **Cz^{9}PhAn**	S11

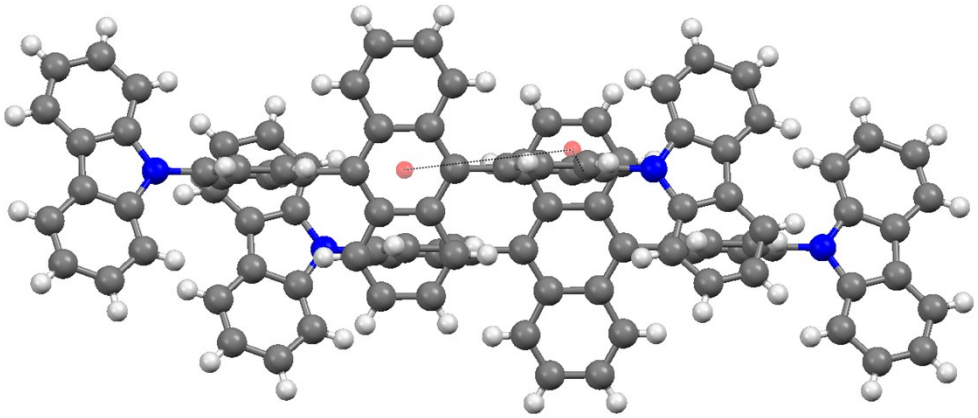

Figure S3. Molecular packing of **Cz^{9}PhAn** from a top view.

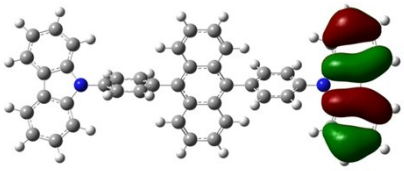 HOMO-3 (-5.91 eV)	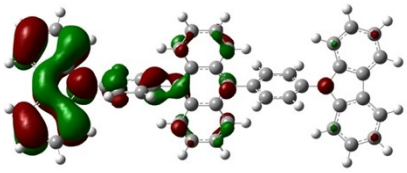 HOMO-2 (-5.52 eV)
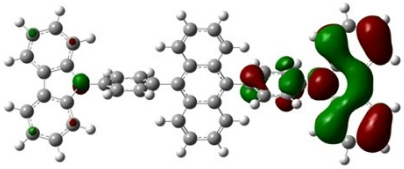 HOMO-1 (-5.50 eV)	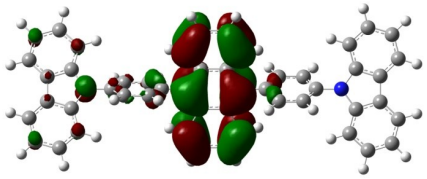 HOMO (-5.34 eV)
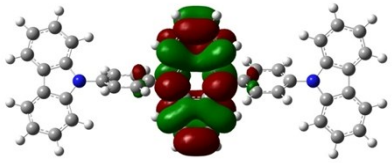 LUMO (-1.87 eV)	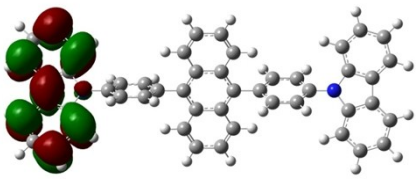 LUMO+1 (-0.84 eV)
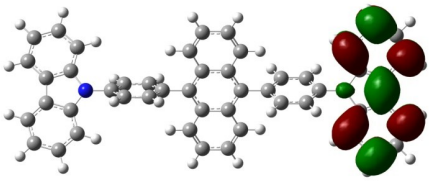 LUMO+2 (-0.84 eV)	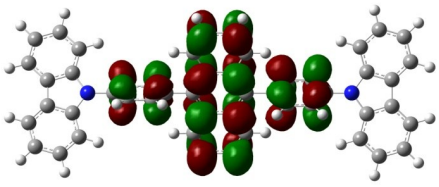 LUMO+3 (-0.67 eV)
